# A dual continuum model of the reasons for use of complementary health approaches among overweight and obese adults: findings from the 2012 NHIS

**DOI:** 10.1186/s12906-018-2404-5

**Published:** 2018-12-20

**Authors:** Dawn M. Upchurch, Linghui Liang, Fuschia M. Sirois

**Affiliations:** 1 Department of Community Health Sciences, UCLA Fielding School of Public Health, 650 Charles Young Drive South, Los Angeles, CA 90095-1772 USA; 2 Department of Psychology, University of Sheffield, Cathedral Court, 1 Vicar Lane, Sheffield, S1 2LT UK

**Keywords:** Complementary therapies, Obesity, Health motivations, Health promotion

## Abstract

**Background:**

Obese and overweight individuals have greater illness and disease burden, but previous findings from the 2002 National Health Interview Survey (NHIS) suggest that they are no more likely to use complementary health approaches (CHA) than those of normal weight. The current study investigates the relationship between weight status and CHA use, and among CHA users, examines differences in reasons for use by weight status. We propose and test a Dual Continuum Model of Motivations for Use of CHA to examine differences in reasons for use by weight status.

**Method:**

Participants were drawn from the 2012 NHIS, a nationally representative sample of civilian, non-institutionalized US adults (*N* = 34,525). Weight status was operationalized by body mass index. CHA use was measured in the past year and was categorized into alternative providers, products, and practices. Among CHA users (*N* = 9307) factors associated with use were categorized as health enhancing or health reactive.

**Results:**

Logistic regression showed overweight and obese individuals were less likely to use alternative providers, products, and practices than normal weight. Multinomial logit regression showed some support that overweight and obese adults were less likely than normal weight persons to use CHA for health-enhancing reasons, and more likely to use for health reactive reasons.

**Conclusions:**

Despite greater health burden, overweight and obese adults are underutilizing CHA, including modalities that can be helpful for health management. The Dual Continuum Model of CHA Motivations shows promise for explicating the diversity of reasons for CHA use among adults at risk for health problems.

**Electronic supplementary material:**

The online version of this article (10.1186/s12906-018-2404-5) contains supplementary material, which is available to authorized users.

## Background

Overweight and obesity continue to be major public health priorities in the US [1]. Obesity is associated with increased risk for type 2 diabetes, metabolic syndrome, cardiovascular disease, and some cancers [2–4], with direct medical costs accounting for 5–10% of annual health care spending [5, 6]. Use of complementary health approaches (CHA) has increased over the past decade [7], with evidence indicating that people use CHA for either health promotion or treatment of existing health problems [8], including chronic illness [9]. Research has found that a range of CHA modalities can be used for weight loss [10], with herbal supplements, relaxation techniques, and massage therapy among the most commonly used CHA associated with obesity [11, 12]. Despite this, and evidence indicating that obesity is associated with greater disease burden and higher use of conventional health care services [13], findings from the 2002 National Health Interview Survey (NHIS) suggest that overweight and obese individuals do not use CHA at greater rates than normal weight individuals [12]. However, given the steady increase and interest and use of CHA in recent years [14], it is unknown whether the findings from 2002 are an accurate reflection of current use of CHA by overweight and obese individuals.

Aside from understanding the relative rates of CHA use among individuals of different weight groups, understanding the motives for CHA use and how they might differ as a function of weight status is also of interest. Most of the research on CHA motives has focused on general motives for use, such as the purpose for using CHA [8] and belief-based motivations [15, 16], rather than how such motives might differ as a function of weight status. Research examining the purpose for CHA use has identified two distinct types of CHA users, those who use CHA for treatment and those who use CHA for health promotion [8]. Building on this work, we propose that CHA for treatment reflects reactive motives, whereas CHA for health promotion reflects health-enhancement motives, and that such motives can be further differentiated according to whether CHA use is directed towards immediate or distal health concerns. We introduce this Dual Continuum Model of Motivations for CHA use to provide a fine-grained, theoretical understanding of the diversity of motivations for using CHA among of normal weight, overweight, and obese individuals.

Early models examining the motives for CHA (formerly known as complementary and alternative medicine, or CAM) took an approach-avoidance motivation perspective, and proposed that CHA users, as compared to non-users, were either “pushed” away from conventional medicine or “pulled” towards CHA [17, 18]. Push factors included negative aspects of conventional care, such as dissatisfaction with treatment, its side effects, and poor doctor-patient relationships [19, 20], whereas pull factors included the positive aspects of CHA, such as perceived benefits of CHA [16, 21], and the congruency with personal beliefs about health and healing [20, 22]. Other conceptualizations took a sociological perspective, and highlighted the importance of medical need for driving use [23]. This socio-behavioral model of CHA use situated medical need as a primary motivator that was embedded within the context of socio-demographic predisposing and enabling factors, such as gender, age, health beliefs, and access or barriers to using CHA, such as income and availability, which operated indirectly to promote use. Research over the past two decades has confirmed the central role of medical need and consistently found those who use CHA have poorer health and a greater number of health problems than non-users [9, 24].

Despite the importance of medical need, recent theory and research into the motives for CHA use has demonstrated that there is a diversity of motives and reasons that go beyond a problem-based, treatment focus. The socio-behavioral wellness model of CHA acknowledges the role of not only medical need but other needs, such as the desire for wellness and health promotion, in motivating use [25, 26]. According to this model, medical needs and lifestyle needs can be distinct or overlap, and thus differentiate CHA users according to whether motives for CHA use are for treatment, wellness, or combined treatment and wellness. This continuum approach to understanding CHA motives has several advantages over previous models as it acknowledges the growing use of CHA use for promoting and maintaining health both in healthy and medically compromised populations [8, 27, 28]. Thus it accounts for motives that span the problem-based and health enhancing spectrum.

Ostensibly, problem-based and health-enhancing motives for CHA use include a temporal dimension, which previous models have not made explicit. Problem-based motives reflect using CHA to address immediate medical needs to ameliorate or manage ongoing or acute health symptoms or conditions. In contrast, health-enhancing motives often focus on using CHA as a means to promote long-lasting positive health, and in this respect are similar to the future-oriented motives that underlie other health behaviors, such as healthy eating and exercise [29, 30]. However, recent evidence suggests that perceived risk for developing disease can also motivate CHA use. Consistent with protection motivation theory [31, 32], beliefs that healthy living can prevent disease in the future differentiated individuals with disease risk from those not at risk among those who used CHA [33]. If we view disease risk as reflecting the potential of a health problem in the future, then CHA use for disease prevention can be considered as a more reactive, problem-based motive that is future rather than present-oriented. Similarly, health-enhancing motives may go beyond those that focus only on health promotion [8] and the maintenance of health into the future, and can include using CHA for the intrinsic rewards it provides. For example, someone may engage in practices such as yoga or meditation because it provides immediate benefits, such as feeling more relaxed, and experiencing an elevated sense of overall well-being (e.g., [34]).

**Fig. 1:**
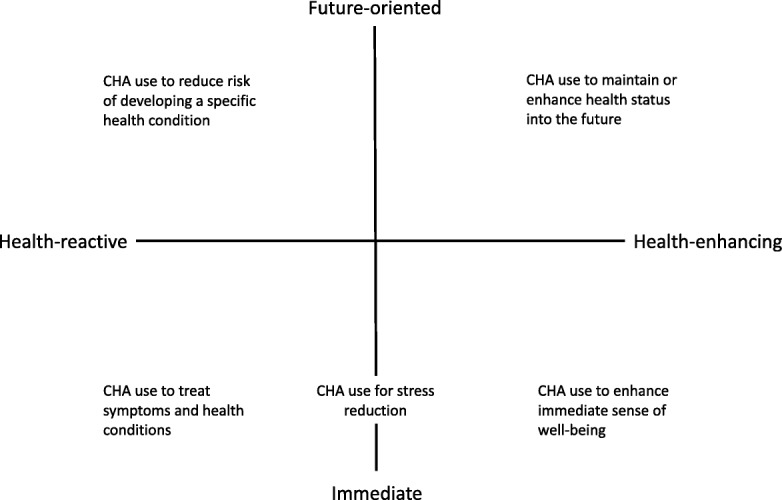
Dual Continuum Model of Motivations for use of Complementary Health Approaches (CHA)

Building on this research and theory, we argue that CHA motives for at-risk groups may be best understood by considering both the continuum of reactive (problem-based) and health enhancing motives, as well as the temporal continuum from immediate to future needs they span. Figure 1 outlines how different motives can be characterised across the intersection of these two continuums. Broadly, those who are at risk for poor health outcomes, because of their current health status, or due to a known risk for the development of a specific disease or set of health problems, can be viewed as being motivated to use CHA for treatment reasons [8], and can be thought of as health reactive. If current health status is poor due to existing chronic conditions, then motives for using CHA are viewed as being driven by immediate medical need, similar to the early socio-behavioral models that were applied to understand CHA use [23]. If, however, health status is generally not compromised, but there is a known risk for the development of disease, then motives for using CHA are viewed as being driven by the need to avoid illness in the future. However, according to this model, CHA use can also arise from motives focused on enhancing health, rather than avoiding or reducing illness. Such motives can span from a focus on using CHA to maintain health into the future, to using CHA because of its intrinsic benefits. Moreover, similar to wellness models of CHA use, motives for CHA use can include a mixture of reactive and health-enhancing motives. For example, using CHA for stress reduction would reflect these mixed motives and fall towards the immediate side of the temporal continuum.

When applied to understanding the motives for CHA use among individuals who are normal weight, overweight, and obese, this model suggests several predictions. Given the known risks of being overweight and obese for health, predictors of CHA use for individuals in these weight categories should include factors associated with more health reactive motives, such as having greater immediate medical needs. In contrast, factors associated with health-enhancing motives, such as engagement in other general health behaviors, would be expected to predict CHA use among those of normal weight, and be less predictive of CHA use among those who are overweight or obese.

### The present study

The purpose of the current study was first to examine the relationships between weight status (normal, overweight, obese) and recent use of CHA to provide an update on whether those who are overweight or obese use CHA at, higher, lower or same rates as normal weight individuals. Then, among recent users, we sought to understand the motives for CHA use among different weight groups using a dual continuum model. Specifically, we hypothesised that the predictors of CHA use among those in at risk weight groups (i.e., overweight and obese) would reflect motives that were health-reactive and focused on immediate health concerns, and rather than on health enhancement. Predictors in this category included having functional limitations, a greater number of health conditions, using CHA for treatment rather than for wellness, and engaging in fewer healthy behaviors. In contrast, we posited that the predictors of CHA use among those in the normal weight group would tend to reflect motives characterised by a focus on health enhancement with a focus on future health. Predictors in this category included engaging in healthy behaviors, and using CHA for wellness rather than for treatment.

## Method

### Participants

The National Health Interview Survey (NHIS) is an ongoing, cross-sectional, in-person household survey of US civilian, non-institutionalized individuals [7]. The current study used data from the 2012 NHIS, the most up-to-date information on CHA use in the US [7]. The NHIS is a multistage probability sample design with clustering, stratification, and oversampling. From each household, a randomly selected adult 18 or over (Sample Adult Core) was selected to complete detailed health and sociodemographic questions (*N* = 34,525). These individuals also completed the Adult Alternative Medicine Supplement. The interviews were conducted using computer-assisted personal interview questionnaires; the Sample Adult Core response rate was 79.7% [7]. In the Alternative Medicine Supplement, adults were asked about their use of over 20 different types of CHA and additional detail was collected on those modalities used. For the current study, the analytic sample included all adults who completed the Alternative Medicine Supplement and had valid data on CHA use (*N* = 33,594). The second analytic sample included only those adults who had used at least one type of CHA in the past year and had valid responses for reason for use (*N* = 9307). The NHIS is publicly available and de-identified, thus this study was exempt from human subject review.

### Measures

#### Outcomes

Following previous research that characterized types of CHA use according to their accessibility, cost, and time commitments [25], and that indicated that CHA users are not homogenous in their choice of modalities [35], CHA modalities were coded into alternative providers, products, and practices. Alternative providers included acupuncturists, biofeedback therapists, chiropractors/osteopaths, energy healers, hypnotists, massage therapists, naturopaths, traditional healers, or other alternative providers as specific by the individual. Alternative products included any one of the many non-vitamin, non-mineral supplements listed in the NHIS (e.g., fish oil, probiotics, Echinacea). Alternative practices included mind-body techniques or movement techniques (e.g., yoga, meditation, tai chi, progressive relaxation, Pilates). The final coding consisted of three dummy variables, representing use (or not) of each type of CHA.

Recent CHA users were asked to specify three of the most important specific CHA modalities used in the past year. For each, they were asked if the modality was used to treat a specific health condition. Any mention of CHA for treatment was counted as a ‘yes’ for treatment only. Use of CHA for wellness only was coded ‘yes’ if there was no mention of use for treatment and the individual responded affirmatively to any of the several wellness items assessed in the NHIS (e.g., general wellness or disease prevention, improve overall health). Last, if users mentioned use of CHA for both treatment and for wellness, they were coded as such.

#### Body mass index (BMI)

BMI was defined as weight in kilograms (kg) divided by height in meters squared (m^2^). It was calculated from individual’s self-report of height and weight. BMI was then coded into the standard categories: under/normal (< 25 kg/m^2^), overweight (25 to < 30 kg/m^2^), and obese (> 35 kg/m^2^).

#### Covariates

To the extent possible, covariates were coded using standard categories presented in other national studies and reports of CHA [7, 36]. Gender was dichotomous. Age was coded ordinally (18–29, 30–49, 50–64, 65+). Race and ethnicity were based on self-report with any mention of Hispanic/Latino given priority (Hispanic/Latino, Non-Hispanic White, Non-Hispanic Black, Non-Hispanic Asian, Non-Hispanic other race). Educational attainment (high school or less, some college, bachelor degree or higher) and annual household income (<$34,999, $35,000-49,999, $50,000-74,999, $75,000-99,999, $100,000+) were coded as ordinal variables. Current marital status was assessed as (never married, married, cohabiting, divorced/widowed, separated). Functional limitations (not limited, any limitation) and mental distress using the Kessler [37] short screen (0–12, 13–24) were coded as dichotomous variables. Coding of the K6 as a dichotomy with the cut points at < 12 and 13+ is recommended because those are the values estimating a threshold for clinical significance of a range of non-specific mental distress [37]. A summary measure of three types of healthy behaviors (not smoking, light to moderate physical activity, adequate leisure-time physical activity) was created by summation (0 to 3).

### Analysis

Cross-tabulations and design-based *F* tests were used for bivariate analysis. Bivariate logistic regression was used to examine the association between BMI status and the use of alternative providers, products, and practices. Among recent CHA users, multinomial logit regression was used to investigate BMI, demographic, health, and health behavior differences in reasons for use (i.e., treatment only, wellness only, combined wellness and treatment). In this analysis, use for treatment only is the referent category. Adjusted odds ratios (AOR) and 95% Confidence Intervals (CI) are presented. All analyses are weighted to adjust for the complex sample design, oversampling, and non-response, and are adjusted to Census controls for sex, age, and race/ethnicity as recommended by NHIS. Thus, results are representative of the US population 18+ in 2012. were conducted with Stata statistical software (SE version 13.1) and used techniques to account for the complex sample design on of the NHIS [38].

## Results

### Descriptive results

Table 1 presents the distributions of demographic, health, and lifestyle characteristics for all individuals and by BMI status for adults 18 and over in 2012. There were significant differences by BMI status for each of the characteristics considered (*p* < .0001). In particular, higher percentages of overweight and obese adults had functional limitations and greater numbers of chronic conditions than those who were under/normal weight. For example, 15.0% of under/normal BMI adults reported 4 or more health conditions compared to 30.9% of adults who were obese. Also, overweight and obese adults reported fewer healthy lifestyle behaviors than those who were under/normal weight.

**Table 1:** Demographic, Health, and Lifestyle Characteristics, All Adults, and by BMI Status, NHIS 2012 (*N* = 33,594)

	Total %	Under/normal %	Overweight %	Obese %	*p*-value
	100.00	36.87	34.55	28.58	
Sex					
Male	45.33	36.22	55.33	45.00	< 0.0001
Female	54.67	65.78	44.67	55.00	
Age					
18–29 years old	19.20	26.96	15.43	13.76	< 0.0001
30–49 years old	32.89	30.73	32.77	35.82	
50–64 years old	25.84	20.37	27.29	31.15	
65+ years old	22.07	21.95	24.52	19.26	
Race/ethnicity					
Hispanic	12.89	10.76	13.88	14.34	< 0.0001
NH-White	69.37	71.28	69.66	66.53	
NH-Black	12.47	9.74	11.97	16.61	
NH-Asian	4.50	7.60	3.74	1.41	
NH-Other	0.80	0.62	0.74	1.11	
Nativity					
Born in US	84.52	82.86	83.25	88.21	< 0.0001
Foreign born	15.48	17.14	16.75	11.79	
Education attainment					
High school and less	39.02	34.72	39.41	44.08	< 0.0001
Some college	31.74	31.34	30.77	33.42	
College and above	29.25	33.94	29.82	22.50	
Annual family income					
$ 0–34,999	40.58	41.15	38.17	42.75	< 0.0001
$ 35,000-49,999	14.19	13.25	14.77	14.70	
$ 50,000-74,999	16.92	16.37	16.99	17.53	
$ 75,000-99,999	10.62	10.13	11.04	10.72	
$ 100,000+	17.77	19.10	19.02	14.29	
Marital status					
Never married	24.10	29.11	20.52	21.97	< 0.0001
Married	43.96	39.68	47.39	45.34	
Divorced/Widowed	25.95	24.98	26.29	26.79	
Cohabiting	5.99	6.24	5.80	5.90	
Functional limitation					
Limited	37.39	30.18	35.06	49.51	< 0.0001
Not limited	62.61	69.82	64.94	50.49	
K6 scores					
0–12	96.82	96.73	97.45	96.17	< 0.0001
13–24	3.18	3.27	2.55	3.83	
Number of chronic conditions					
0	32.09	40.41	31.35	22.26	< 0.0001
1	20.98	22.76	21.04	18.63	
2–3	25.20	21.81	26.35	28.18	
4+	21.73	15.02	21.27	30.93	
Healthy behavior index					
0	5.82	6.49	5.32	5.54	< 0.0001
1	39.57	39.34	37.68	42.13	
2	43.67	41.59	45.50	44.13	
3	10.95	12.57	11.50	8.20	

Notes: *p*-values for bivariate design-based *F* test. NH: Non-Hispanic. All percentages are weighted, see text for additional information

### CHA use as a function of weight status

Table 2 shows the prevalence of recent use of providers, products, and practices by BMI status and results from three logistic regression models. For each type of CHA, overweight and obese individuals had significantly lower use than those who were under/normal weight with obese persons having the lowest use. For example, 19.0% of under/normal weight reported using practices in the past year, 11.1% of obese used them. These bivariate findings were largely confirmed by the logistic regression results controlling for demographics, health and lifestyle factors. Compared to under/normal weight people, those who were overweight or obese had significantly lower odds of using CHA providers, products, or practices (except for overweight users of providers).

**Table 2:** Prevalence of Use of Alternative Providers, Products, and Practices and Logistic Regression Results for Each, by BMI Status, NHIS 2012 (*N* = 33,594)

	%	*p*-value^1^	AOR	95% CI	*p*-value^2^
Providers	15.67				
Under/Normal	17.10	<.0001	1.00		
Overweight	15.55		0.95	(0.88, 1.04)	0.267
Obese	13.97		0.78	(0.71, 0.87)	<.0001
Products	19.22				
Under/Normal	20.46	.0006	1.00		
Overweight	18.88		0.89	(0.82, 0.97)	0.010
Obese	18.02		0.78	(0.71, 0.85)	<.0001
Practices	14.65				
Under /Normal	18.94	<.0001	1.00		
Overweight	12.94		0.78	(0.71, 0.86)	<.0001
Obese	11.19		0.59	(0.53, 0.65)	<.0001

Note: ^1^ *p*-values for bivariate design-based *F* test. ^2^ *p*-value for multivariate regressions. Results from 3 separate multivariate models that also included gender, age, race/ethnicity, nativity status, educational attainment, income, marital status, functional limitations, K6 score, number of chronic conditions, and number of health behaviors. All percentages and analyses are weighted, see text for additional information

### Motivations for CHA use among different weight groups

Table 3 considers only CHA users and examines differences in reasons for use based on BMI status, and presents both bivariate and multinomial logit regression results. There were significant differences in reasons for use by BMI status, with higher percentages of overweight and obese reporting CHA use for treatment only or for both wellness and treatment combined, and lower percentages reporting CHA use for wellness only relative to under/normal weight people. Over half of under/normal weight individuals reported using CHA for wellness only compared to 39.8% of obese individuals. Regression results show that, compared to normal weight, overweight and obese individuals had lower odds of reporting CHA use for wellness only versus treatment only, although the effects were significant only for overweight individuals. There were no significant differences in CHA use for combined wellness and treatment versus treatment alone as a function of BMI status.

**Table 3:** Prevalence of CHA Use for Treatment Only, Wellness Only, and Both Wellness and Treatment, by BMI Status (Panel 1), and Multinomial Logit Regression Results Comparing Characteristics According to Reason for Use (Panel 2), NHIS 2012 (*N* = 9307)

Panel 1				
	Total %	Under/Normal %	Overweight %	Obese %
Reason for use				
Treatment only	11.68	9.38	13.06	13.54
Wellness only	45.86	51.19	44.01	39.75
Both wellness and treatment	42.46	39.43	42.94	46.70
Panel 2				
	Wellness only vs. treatment only AOR	95% CI	Both wellness and treatment vs. treatment only AOR	95% CI
BMI Status				
Under/Normal	1.00		1.00	
Overweight	0.82*		0.87	
Obese	0.85+		0.84+	
Health and Lifestyle				
Functional limitations	0.58***		1.10	
K6	1.66*		1.55+	
Health conditions	0.90***		1.06*	
Healthy behaviors	1.13*		1.25***	

Note: *p*-value for bivariate design-based *F* test. *p* < .00001. Sample size includes all recent CHA users who mentioned up to three most important CHA modalities and responded to reasons for using each. Results from multinomial logit regression model that also included gender, age, race/ethnicity, nativity status, educational attainment, income, and marital status. All analyses weighted, see text for additional information

+ *p* < .1; * *p* < .05; ** *p* < .01; *** *p* < .001

Those with functional limitations or more health conditions had lower odds of using CHA for wellness only versus treatment only. Also, those with mental distress or more healthy behaviors had higher odds of CHA use for wellness only versus treatment only. Lastly, those with more health conditions or healthy behaviors had higher odds of using CHA for both wellness and treatment versus treatment alone (relative to those with fewer health conditions).

## Discussion

The current study provides an updated and theory driven perspective on the use of CHA with respect to weight status, and the diversity of motives that distinguish different weight groups in a nationally representative sample of US adults. In contrast to previous findings with the 2002 NHIS [8], the current analysis of 2012 NHIS found that individuals who are overweight or obese were less likely to use alternative providers, products, or practices compared to normal weight adults. Among recent users of CHA, we found some support for the proposition that overweight and obese adults were less likely to use CHA for health-enhancing reasons and more likely to use CHA for health-reactive reasons compared to normal weight adults. Supporting our hypotheses, we found that overweight and obese individuals reported a greater number of health conditions and functional limitations than normal weight individuals, and that these indictors of poor health status were predictors of using CHA for treatment as opposed to wellness. We also found that those with more chronic health conditions or functional limitations were less likely, and those who engage in a greater number of healthy behaviors more likely, to use CHA for health-enhancing reasons. Taken together, these findings suggest Dual Continuum Model of Motivations for Use of CHA shows promise for understanding the different motivations and reasons for using CHA among individuals at risk for poor health. In addition, our findings suggest that overweight and obese adults are underutilizing CHA, including types which have the potential to reduce health burden (see Additional file 1: Table S1).

The current findings that overweight and obese adults have lower rates of use were in line with other research on CHA use and BMI status [12, 39]. However, we extend this research by taking a more refined view of CHA use and characterizing CHA types as alternative providers, products, and practices. The significant differences by weight status did not change when functional limitations, health conditions, and lifestyle behaviors were taken into account, suggesting that other explanations beyond weight-related health and lifestyle differences must be considered. Additionally, overweight and obese adults report lower use for all but one specific CHA modality, and obese adults have lowest use of almost every CHA type. In particular, relative to normal weight adults, those who are obese had much lower rates of use of movement modalities (e.g., yoga, tai chi) and some others involving body exposure and manipulation (e.g., massage, acupuncture) by a provider. There is ample evidence supporting bias and stigmatization with respect to body size, including within the health care system, and often resulting in avoidance of care (e.g., [40–42]), and it is possible this could be a contributing factor for lower use of these CHA types. In any event, our findings point to the need to further examine reasons for lower use, to explicitly consider body size bias as a potential hindrance of use, especially body movement and manipulation types of CHA. Importantly, it is just those CHA modalities (e.g., yoga, massage) that can potentially be helpful in obesity-related symptoms such as pain that heavier persons are less likely to use.

Our findings also underscore the importance of shifting motives for CHA use from immediate health reactive to health enhancing among individuals in at risk weight groups, and the public health implications of doing so. Consistent with the Dual Continuum Model of Motivations for Use of CHA, we found some support that overweight and obese individuals are less likely to use CHA for health-enhancing reasons and more likely to use for health-reactive reasons. Overweight and obese CHA users report higher rates of use for treatment only, and lower for wellness only compared to normal weight users. These findings are partially confirmed in the multivariate analysis showing lower odds of use for wellness only. In addition, individuals with more health problems appear to be using CHA for more health-reactive reasons and those with healthier lifestyles for more health-enhancing. It may well be that users who are healthier consider and use CHA as part of a health-enhancing and wellness lifestyle [8, 43]. Moreover, combined use was endorsed by both users with more health conditions and greater number of healthy behaviors, suggesting CHA can be used to maintain or improve quality of life in the context of existing health problems.

The current findings provide preliminary support for the utility of the Dual Continuum Model of Motivations for Use of CHA for understanding the different motives for CHA use among individuals with different weight status. Further work is needed to verify if the motive continuums – health-reactive versus health-enhancing, and immediate versus future needs – are useful for explicating the diversity of motives for other at risk groups who may turn to CHA for their health needs. For example, future work could assess the extent to which CHA is used as a reaction to the threat of perceived future health problems, as emerging research suggests that prevention motives for CHA use figure prominently among those at risk for poor heart health [33].

The findings from the current study, though novel, should be considered within the context of several limitations. First, the NHIS is a cross-sectional study therefore we cannot establish the causal nature of the relationships examined. Second, the estimates for overweight and obesity in these data are lower than in studies in which participants are weighed and measured because NHIS uses self-reported information. Third, although there is substantial detail in the NHIS with respect to reasons for CHA use, to more fully test the proposed Dual Continuum Model of Motivations for Use of CHA, a more comprehensive set of measures that more closely align with our constructs of health-enhancing and health-reactive motives is needed. Nonetheless, a significant strength of the NHIS is that it is a large, nationally representative study that includes the most comprehensive and most recent assessment of CHA use in the US. In addition, we examined CHA use as a function of different groups of modalities, which provided a more nuanced view of differences among weight groups.

## Conclusions

Americans are using CHA for a variety of reasons, and there is growing evidence that they are increasingly including them as part of a health-promoting and wellness lifestyle [8, 16, 28]. The growing scientific evidence-base for several modalities and the increase in integrative care within conventional medical settings points to the need to continue to understand the potentially complex motivations for CHA use and to provide opportunities and outreach to those groups, including overweight and obese individuals, with unmet needs. From the perspective of the Dual Continuum Model of Motivations for Use of CHA, our findings suggest that both immediate and future health concerns may have to be considered when understanding and addressing these needs.

## Additional file

Additional file 1: **Table S1.** Rank Order of Specific CHA Modalities, Total, and by BMI Status, NHIS, 2012 (*N* = 11,516). (DOCX 16 kb)

## Abbreviations

AOR: Adjusted Odds Ratio
BMI: Body mass index
CHA: Complementary health approaches
CI: 95% Confidence Interval
NHIS: National Health Interview Survey
US: United States
