# Supplementary material for: A dual continuum model of the reasons for use of complementary health approaches among overweight and obese adults: findings from the 2012 NHIS
Source: BMC Complement Altern Med. 2018 Dec 20;18:339. doi: 10.1186/s12906-018-2404-5 (PMC6302527; doi:10.1186/s12906-018-2404-5)
Supplement: Supplementary file 1 — Table S1. Rank Order of Specific CHA Modalities, Total, and by BMI Status, NHIS, 2012 (N = 11,516). (DOCX 16 kb) [file 12906_2018_2404_MOESM1_ESM.docx]

| Table S1. *Rank Order of Specific CHA Modalities, Total, and by BMI Status, NHIS, 2012 (N = 11,516)* | | | | | | |
| --- | --- | --- | --- | --- | --- | --- |
| Rank | **Specific CHA modality** | **%** | | | | |
|  |  | **Total** | **Low/Normal** | **Overweight** | **Obese** | ***p*-value** |
| 1 | Multi-vitamin or multi-mineral | 53.63 | 55.63 | 54.41 | 50.12 | <0.0001 |
| 2 | Herbal or other non-vitamin supplements | 18.43 | 19.52 | 18.16 | 17.34 | 0.0024 |
| 3 | Yoga, Tai Chi or Qi Gong | 10.26 | 15.22 | 8.53 | 5.97 | <0.0001 |
| 4 | Chiropractic or osteopathic manipulation | 9.36 | 9.54 | 9.66 | 8.75 | 0.1330 |
| 5 | Massage | 9.19 | 10.26 | 8.96 | 8.10 | <0.0001 |
| 6 | Meditation, guided imagery or progressive relaxation | 5.15 | 6.07 | 4.83 | 4.35 | <0.0001 |
| 7 | Special diet | 3.16 | 3.41 | 2.73 | 3.37 | 0.0219 |
| 8 | Homeopathy | 2.35 | 3.10 | 1.87 | 1.96 | <0.0001 |
| 9 | Movement techniques: Feldenkrais, Pilates, Trager psychophysical integration, or Alexander technique | 2.27 | 3.84 | 1.51 | 1.16 | <0.0001 |
| 10 | Acupuncture | 1.75 | 2.36 | 1.66 | 1.08 | <0.0001 |
|  | | | | | | |

Note: *p*-values for bivariate design-based *F* test.
